# Clinicopathological analysis of colorectal cancer: a comparison between emergency and elective surgical cases

**DOI:** 10.1186/1477-7819-11-133

**Published:** 2013-06-11

**Authors:** Sam Ghazi, Elisabeth Berg, Annika Lindblom, Ulrik Lindforss

**Affiliations:** 1 Department of Laboratory Medicine, Division of Pathology, Karolinska Institutet, Karolinska University Hospital at Huddinge, Stockholm S-14186, Sweden; 2 Department of Learning, Informatics, Management and Ethics (LIME), Karolinska Institutet, Medical Statistics Unit, Stockholm S-171 77, Sweden; 3 Department of Molecular Medicine and Surgery, Karolinska Institutet, Karolinska University Hospital at Solna, Stockholm S-17176, Sweden; 4 Department of Clinical Science, Innovation and Technology, Division of Surgery, Karolinska Institutet, Karolinska University Hospital at Huddinge, Stockholm S-14186, Sweden

**Keywords:** Emergency surgery for colorectal cancer, Prognostic factors, Histopathology of colorectal cancer

## Abstract

**Background:**

Approximately 15 to 30% of colorectal cancers present as an emergency, most often as obstruction or perforation. Studies report poorer outcome for patients who undergo emergency compared with elective surgery, both for their initial hospital stay and their long-term survival. Advanced tumor pathology and tumors with unfavorable histologic features may provide the basis for the difference in outcome. The aim of this study was to compare the clinical and pathologic profiles of emergency and elective surgical cases for colorectal cancer, and relate these to gender, age group, tumor location, and family history of the disease. The main outcome measure was the difference in morphology between elective and emergency surgical cases.

**Methods:**

In total, 976 tumors from patients treated surgically for colorectal cancer between 2004 and 2006 in Stockholm County, Sweden (8 hospitals) were analyzed in the study. Seventeen morphological features were examined and compared with type of operation (elective or emergency), gender, age, tumor location, and family history of colorectal cancer by re-evaluating the histopathologic features of the tumors.

**Results:**

In a univariate analysis, the following characteristics were found more frequently in emergency compared with elective cases: multiple tumors, higher American Joint Committee on Cancer (AJCC), tumor (T) and node (N) stage, peri-tumor lymphocytic reaction, high number of tumor-infiltrating lymphocytes, signet-ring cell mucinous carcinoma, desmoplastic stromal reaction, vascular and perineural invasion, and infiltrative tumor margin (*P*<0.0001 for AJCC stage III to IV, N stage 1 to 2/3, and vascular invasion). In a multivariate analysis, all these differences, with the exception of peri-tumor lymphocytic reaction, remained significant (*P*<0.0001 for multiple tumors, perineural invasion, infiltrative tumor margin, AJCC stage III, and N stage 1 to 2/3).

**Conclusions:**

Colorectal cancers that need surgery as an emergency case generally show a more aggressive histopathologic profile and a more advanced stage than do elective cases. Essentially, no difference was seen in location, and therefore it is likely there would be no differences in macro-environment either. Our results could indicate that colorectal cancers needing emergency surgery belong to an inherently specific group with a different etiologic or genetic background.

## Background

Colorectal carcinoma (CRC) is the third most common form of cancer worldwide. Around 15 to 30% of CRCs present as a surgical emergency, with the most common causes being obstruction (78%), perforation (10%), or bleeding (4%) [1,2]. Rectal cancers seldom present as an emergency (5.9%), whereas this is much more likely with colon cancers (21.7%) [2]. The left colon and the sigmoid are the most common sites of tumor obstruction, but the risk for obstruction seems to be highest at the splenic flexure [3,4]. The sigmoid and the cecum have been reported to be the most common sites of perforation [5]. Perforation can occur either at the site of the tumor or proximal to it, and is a serious condition that, apart from the risk of tumor cells seeding, can result in generalized peritonitis or abscess formation.

Patients undergoing acute surgery are generally older than elective cases (mean age 68.6 and 66.3 years, respectively) and some studies have shown a female predominance (50.3% and 43%, respectively). Both young (<40 years) and old (>80 years) patients with CRC more often present as an emergency, probably because both groups are at risk of having their symptoms regarded with indifference [2].

Many studies report a poorer outcome for patients who undergo emergency surgery, both during their initial hospital stay and for their long-term survival [1,2,5-7]. Emergency surgery for CRC is associated with a higher risk for metastatic disease, possibly because of occult liver metastases [3,6,8], although such cancers do not necessarily show a higher rate of local recurrence [3]. In one study, the 5-year overall survival rate following emergency surgery was 39.2%, compared with 64.7% for elective cases [2], and a median survival time of 59 months for emergency compared with 82 months for elective surgery has also been reported [7]. Advanced tumor pathology and tumors with unfavorable histologic features may be reasons for this difference in outcome.

Patients undergoing emergency surgery tend to have more advanced cancers (American Joint Committee on Cancer (AJCC) stages III and IV), with more tumor (T)3 and T4 tumors and more node (N)1 and N2 cases compared with electively managed patients [2,5]. According to some studies, on a stage-for-stage analysis, the survival rates remain lower for emergency cases even after sub-stratification for factors such as lymph-node status and presence of extramural lymphovascular invasion [2,5]. R1 resections are also more common among cases presenting as a surgical emergency (10% versus 1%) [7].

Many studies have found no difference in the morphological profiles of emergency and elective CRCs [3,8-10]. However, in one study [7], extramural venous invasion was more common in emergency cases (20% versus 6%), and the survival of patients with obstructive CRC has been linked to the presence of a mucinous tumor [11]. Although Abdelrazeq *et al*. [12] found that perforated tumors were more likely to present with distant metastases, they also found that these tumors were less likely to be poorly differentiated and had less lymph-node involvement. These findings are difficult to interpret, but could indicate that there is a histologic explanation for the poorer surgical outcome in tumors presenting as an emergency compared with elective cases.

In a previous study [13], we found that there is a ‘right-sided’ type of colon cancer, with features such as larger tumor size, higher T and AJCC stage, poor differentiation, and circumscribed tumor margin. The ‘left-sided’ type of colon cancer and rectal cancer share similar features, with smaller tumor size, lower T and AJCC stage, and infiltrating tumor margin.

The aim of this retrospective study was to compare the clinical and pathologic profiles of CRC cases, treated surgically either as an emergency or electively, with gender, age group, tumor location, and family history of CRC.

## Methods

### Ethics approval

This study was approved by the local Ethics Committee at Karolinska Institutet (no. KI Dnr 02–489), and informed consent was obtained from all participants.

### Patients

University clinics, district general hospitals, and general hospitals participated in the study. In total, 2573 consecutive patients treated surgically for CRC at the eight hospitals in Stockholm County between 2004 and 2006 were assessed for the study. Of these patients, 308 died before being asked to participate. Other reasons for exclusion were that patients were too ill or too old. Finally, 1205 patients (46.8% of the initial total) were included in the study. In 976 cases, tumors were available for re-evaluation. Medical records containing information on type of operation could be found for all but two cases.

Recruitment of patients was carried out either by the individual surgeons after surgery or by us, using a list provided by the Stockholm-Gotland Oncology Center where all cancers in Stockholm County are registered. A letter was sent to each patient with information on the study and a request that they participate. All patients who showed interest in participating were contacted over the telephone for informed consent and thereafter included in the study. A family history of cancer was taken from all study participants, and all CRC diagnoses in the family were verified by medical records or death certificates.

In this study, an emergency case was defined as a patient who underwent emergency CRC surgery because of perforation, obstruction, or bleeding, regardless of the time elapsed from hospital admission to operation. All other cases were considered elective. Most cases were discussed at a post-operative multidisciplinary consensus conference, where they were classified as having undergone emergency or elective surgery. Perforation was defined as pneumoperitoneum on preoperative radiography. Obstructive tumors were defined as tumors causing ileus, regardless of whether the occlusion was partial or total. Bleeding was defined as blood loss from the tumor causing such severe anemia that the patient had to be treated surgically.

Data on gender, age, and tumor location were obtained for all cases. It was possible to obtain information about family history of CRC in 962 cases. Familial CRC was defined as patients with one or more first-degree or second-degree relatives with CRC, who did not fulfill the Amsterdam criteria for Lynch syndrome (LS) or who had evidence of heredity for familial adenomatous polyposis (FAP). Eight patients with LS were found in our sample, but no case of FAP.

### Pathology

All tumors were re-evaluated using a standardized protocol that included information on patient gender, age at operation, name of hospital and pathology department, date of diagnosis, date of re-evaluation, and name of re-evaluating pathologist. Information on tumor location and multiple co-existing tumors was gathered from the original pathology report and from the Stockholm-Gotland Oncology Center Register.

All macromorphologic parameters, including tumor size in three dimensions, were obtained from the original pathology report (all CRCs in Sweden are examined in a standard manner in accordance with a nationwide protocol). The number of positive and negative lymph nodes and the number of blocks taken (including large sections) were noted.

In all 976 cases, slides stained with hematoxylin and eosin (H&E) were obtained from the participating pathology departments. In one case, only biopsy tissue could be retrieved, and in a further 24 cases, the specimen was taken at polypectomy or local resection. All cases were re-evaluated by one experienced gastrointestinal pathologist (SG).

The micromorphologic parameters assessed were tumor grade, stage, medullary features, mucin production, mucinous type, Crohn-like peri-tumor lymphocytic reaction, tumor- infiltrating lymphocytes (TILs), desmoplasia, tumor necrosis, vascular invasion, perineural growth, co-existing polyps, budding, and type of tumor margin. The exact definition of such features and methods of assessing them has been outlined in detail in our previous report [13]. Because of preoperative radiotherapy, rectal cancers were omitted from the analysis of necrosis, desmoplastic reaction, and budding.

### Statistical analysis

Analyses were made using SPSS Statistics (version 20 for Windows; SPSS/IBM, Chicago, IL, USA). The associations between clinical and pathologic features and type of surgery, gender, age group, tumor location, and family history were examined using univariate, multiple binary, and multinomial logistic regression analyses for categorical outcomes, and linear regression analysis for continuous outcomes. The associations between type of surgery and gender, age group, tumor location, family history were examined using similar analyses. Results are presented as odds ratios (ORs) from logistic regression, and as regression coefficients (*b*) from linear regression. The significance level was set at *P*<0.05.

In addition, factor analysis (extracting factors using principal components analysis) with varimax rotation was performed to form a concise description for all variables included in the study. This analysis seeks a few underlying dimensions (factors) that account for patterns of variation among the variables in the study, in this case the clinical and pathologic parameters such as type of surgery, gender, age, tumor location, family history, and morphologic features. Variables with a loading of greater than 0.40 are usually applied as meaningful factor loadings. If a variable has a meaningful loading on more than one component, that variable should be ignored in the interpretation.

## Results

### Descriptive data

The total number of patients examined was 976, of whom 53 had multiple co-existing cancers. Most (86.6%) of cases were elective (n = 845) and 13.2% were emergency (n = 129) cases. In two cases, the type of operation could not be defined. For all but two of the emergency cases, the indication for surgery was found in the medical records: 16% (n = 21) because of perforation, 73% (n = 94) because of obstruction, and 4% (n = 5) because of bleeding. In seven cases, the reason for the emergency surgery was not stated.

Of the 976 patients, 52.6% were men (n = 513) and 47.4% were women (n = 463). Mean age was 69.2 years (median 70.0 years, range 28–95 years). The majority (77.4%) of cases were sporadic CRCs (n = 755) and 20.5% were familial (n = 200). In 13 cases, there was no information on any family history. Eight cases were known to have LS based on the Amsterdam criteria or screening; seven of these were in the elective group and one in the emergency group.

**Figure 1:**
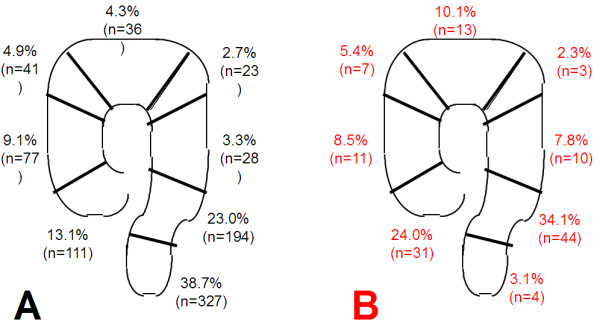
**Percentages of tumors at each location for the elective and emergency surgery cases.** The percentage of tumors was calculated for each location (cecum, ascending colon, hepatic flexure, transverse colon, splenic flexure, descending colon, sigmoid, and rectum) for (**A**) the elective and (**B**) emergency surgery cases. Cases in the appendix were omitted from both groups (n = 1 and n = 2 respectively).

The tumor localization distributions for the elective and the emergency group are shown in Figure 1.

### Comparison by univariate analysis of clinical and pathologic features in the elective and the emergency surgery cases

As shown in Table 1, the emergency cases were significantly more likely to have multiple tumors (OR = 2.816, *P* = 0.001), vascular invasion (OR = 2.086, *P*<0.0001), perineural invasion (OR = 2.032, *P* = 0.001), and an infiltrative tumor margin (OR = 1.666, *P* = 0.008). There was no difference in mucin production. However, when the mucinous tumors of both groups were compared, those in the emergency group were more likely to have a signet-ring cell component (OR = 3.267, *P* = 0.001). Compared with the elective patients, the emergency patients had more tumors of AJCC stages II to IV (*P*<0.0001 for stages III and IV) than stage I. They also had more T stage 3 and 4 tumors and more N-stage 1 and 2/3 tumors (*P*<0.0001 for both N1 and N2/3). Desmoplasia was also more common in the emergency group (OR = 2.110, *P* = 0.03), as was a Crohn-like lymphocytic reaction (OR = 1.554, *P* = 0.03). In contrast to elective cases, emergency cases were less likely to have greater than 30 TILs per 10 high-power fields (HPFs) (OR = 0.551, *P* = 0.04).

**Table 1:** Univariate comparison of clinical and pathologic features in cases of colorectal cancer treated surgically on electively and as an emergency

**Feature**	**Elective** **(n = 845)**	**Emergency** **(n = 129)**	**OR/diff**^**a**^	***P*-value for OR/diff**^**a**^
> 1 tumors, %	4.5	11.7	2.816	0.001^b^
Mean tumor diameter, mm	47	48	0.97	0.68
AJCC stage, %				
I	21.4	3.9	RC	
II	39.1	31.2	4.382	0.002^b^
III	35.1	57.0	8.900	<0.0001^b^
IV	4.3	7.8	9.889	<0.0001^b^
T, %				
1	7.7	2.3	RC	
2	20.0	4.7	0.771	0.72
3	60.5	68.8	3.747	0.03^b^
4	11.7	24.2	6.818	0.002 ^b^
N, %				
0	61.5	36.0	RC	
1	19.7	33.6	2.912	<0.0001^b^
2 or 3	18.8	30.4	2.757	<0.0001 ^b^
Proportion of poorly differentiated tumors, %^c^	10.9	14.7	1.412	0.21
Mucin production, %				
0%	62.4	56.6	RC	
0 to 50%	24.2	25.6	1.168	0.49
>50%, mucinous type	13.4	17.8	1.468	0.14
Mucin type, if mucinous, %				
Extracellular	88.3	69.8	RC	
Signet-ring-type component	11.7	30.2	3.267	0.001^b^
Crohn-like lymphocytic reaction, %	59.8	69.8	1.554	0.03^b^
TILs				
≤30/10 HPFs	80.7	88.4	RC	
>30/10 HPFs	19.3	11.6	0.551	0.04^b^
Desmoplasia,^d^ %	82.6	90.9	2.110	0.03^b^
Necrosis,^d^ %	66.3	61.7	0.816	0.33
Vascular invasion, %	22.2	37.3	2.086	<0.0001^b^
Perineural invasion, %	16.4	28.6	2.032	0.001^b^
Medullary type, %	4.8	3.2	0.646	0.41
Budding,^d^ %	40.0	45.5	1.248	0.28
Tumor margin, %				
Circumscribed	54.5	41.9	RC	
Infiltrative	45.5	58.1	1.666	0.008^b^

*AJCC* American Joint Committee on Cancer, *HPF* high-power field, *N* node, *RC* reference category, *T* tumor, *TILs* Tumor-infiltrating lymphocytes.

^a^Emergency versus elective. Odds ratio except for tumor diameter where difference (mm) is stated.

^b^Significantly different.

^c^In major tumor component.

^d^Rectal cancers were omitted from the analysis of necrosis, desmoplasia, and budding because of preoperative radiotherapy.

### Comparison of clinical and pathologic features in relation to the nature of surgery (emergency/elective), gender, age group, tumor location, and family history by multivariate analysis

In this comparison (Tables 2 and 3), together with gender, age group, tumor location, and family history, the nature of surgery remained a significant factor for multiple tumors, vascular invasion, perineural invasion, tumor margin, mucin type, AJCC stage, T and N stage and TILs. The highest level of significance (*P*<0.0001) was seen for multiple tumors (OR = 3.154), perineural invasion (OR = 2.500), an infiltrative tumor margin (OR = 2.452), AJCC stage III versus I (OR = 6.932), N1 vs N0 (OR = 3.186), and N2/3 versus N0 (OR = 2.679). Crohn-like lymphocytic reaction was the only feature where nature of surgery had no significance.

**Table 2:** **Multivariate analysis of clinical and pathologic features in relation to nature of surgery, gender, age group, tumor location, and family history**^**a**^

	**>1 tumor (yes vs. no)**	**Tumor diam.** **(mm)**	**Differentiation (poor vs. other)b**	**Mucin type (signet-ring component vs. extracellular)**	**Crohn-like reaction (yes vs. no)**	**TILs (>30/HPF vs. ≤30/10 HPF)**	**Desmoplasia (yes vs. no)**	**Necrosis (yes vs. no)**	**Vascular (yes vs. no)**	**Perineural (yes vs. no)**	**Medullary type (yes vs. no)**	**Budding (yes vs. no)**	**Tumor margin (infiltrative vs. circumscribed)**
Type of surgery													
Elective, n = 845	RC	46.89	–	RC	–	RC	RC	–	RC	RC	–	–	RC
Emergency, n = 129	3.154	−3.7	–	3.136	–	0.375	1.932	–	2.086	2.500	–	–	2.452
*P*	<0.0001^c^	0.11	–	0.001^c^	–	0.001^c^	0.04^c^	–	0.001^c^	<0.0001^c^	–	–	<0.0001^c^
Gender													
Male	–	–	–	–	–	–	–	–	–	–	–	RC	–
Female												1.417	–
*P*	–	–	–	–	–	–	–	–	–	–	–	0.03^c^	–
Age group													
>75 years	RC	47.85	–	–	–	–	–	–	–	–	–	–	RC
60 to 75 years	0.732	0.59	–	–	–	–	–	–	–	–	–	–	1.123
	*P*	0.28	0.73	–	–	–	–	–	–	–	–	–	–	0.46
<60 years	NPC^d^	5.69											1.928	
*P*		0.009^c^	–	–	–	–	–	–	–	–	–	–	0.001	
Localization														
Right colon	–	56.42	RC	–	RC	RC	–	–	–	RC	RC	–	RC	
Left colon		−12.67	0.224		0.587	0.278				1.005	0.114		1.119	
*P*	–	<0.0001^c^	<0.0001^c^	–	0.005^c^	<0.0001^c^	–	–	–	0.98	<0.0001^c^	–	0.51	
Rectum		−18.53	0.302		0.123	0.168				1.812	NPC^d^		2.902	
*P*	–	<0.0001^c^	<0.0001^c^	–	<0.0001^c^	<0.0001^c^	ND^e^	ND^e^	–	0.005^c^		ND^e^	<0.0001^c^	
Family history														
Sporadic	–	–	–	–	–	–	–	–	–	–	–	–	–	
Familial							2.028				0.318			
*P*	–	–	–	–	–	–	0.03^c^	–	–	–	0.03^c^	–	–	
Nagelkerke *R*^2^^f^	0.103	0.099	0.083	0.048	0.223	0.140	0.028	–	0.020	0.034	0.233	0.010	0.101	

*AJCC* American Joint Committee on Cancer, *HPF* high-power field, *N* node, *NPC* not possible to calculate, *ND* not done, *RC* reference category, *T* tumor, *TILs* tumor-infiltrating lymphocytes.

^a^Data are presented as odds ratios for all features except tumor diameter, for which mean diameter and difference *b* (mm) are stated.

^b^In major tumor component.

^c^Significant.

^d^It was not possible to calculate odds ratio because there were no multiple tumors in the <60 years age group and only one rectal cancer with medullary features.

^e^Rectal cancers were omitted from the analysis of necrosis, desmoplasia and budding because of preoperative radiotherapy.

^f^Or adjusted *R*^2^.

**Table 3:** **Multivariate analysis of clinical and pathologic features in relation to nature of surgery, gender, age group, tumor location, and family history**^**a,b**^

	**AJCC stage (II, III, and IV vs. I)**		**T stage (T2, T3, and T4 vs. T1)**		**N stage (N1 and N2/N3 vs. N0)**		**Mucin production (0 to 50% and >50% vs. 0%)**	
Type of surgery								
Elective (n = 845)	RC		RC		RC		RC	
Emergency (n = 129)	II	2.854	T2	0.838	N1	3.186	0 to 50%	1.052
		*P* = 0.03^c^		*P* = 0.81		*P*<0.0001^c^		*P* = 0.83
	III	6.932	T3	2.853	N2/N3	2.679	>50%	1.093
		*P*<0.0001^c^		*P* = 0.09		*P*<0.0001^c^		*P* = 0.75
	IV	5.019	T4	4.056				
		P=0.006 ^c^		P=0.03 ^c^				
Gender								
Male	RC		RC		RC		RC	
Female	II	0.863	T2	1.369	N1	0.761	0-50%	0.986
		*P* = 0.44		*P* = 0.29		*P* = 0.11		*P* = 0.93
	III	0.782	T3	1.006	N2/N3	0.916	>50%	0.694
		*P* = 0.20		*P* = 0.98		*P* = 0.62		*P* = 0.07
	IV	0.844	T4	1.494				
		*P* = 0.66		*P* = 0.21				
Age group								
>75 years	RC		RC		RC		RC	
60 to 75 years	II	1.538	T2	1.017	N1	0.790	0-50%	0.713
		*P* = 0.04 ^c^		*P* = 0.96		*P* = 0.22		*P* = 0.06
	III	1.414	T3	1.361	N2/N3	1.462	>50%	1.229
		*P* = 0.10		*P* = 0.30		*P* = 0.07		*P* = 0.39
	IV	1.829	T4	1.842	–	–	–	–
		*P* = 0.13		*P* = 0.09	–	–	–	–
					
<60 years	II	1.830	T2	1.265	N1	1.117	0 to 50%	0.658
		*P* = 0.04^c^		*P* = 0.61		*P* = 0.65		*P* = 0.09
	III	2.019	T3	1.998	N2/N3	1.919	>50%	1.967
		*P* = 0.01^c^		*P* = 0.10		*P* = 0.01^c^	–	P=0.02 ^c^ –
	IV	3.203	T4	3.155	–	–	–	–
		*P* = 0.02^c^		*P* = 0.02^c^	–	–	–	–
Localization								
Right colon	RC		RC		RC		RC	
Left colon	II	0.513	T2	0.333	N1	1.168	0 to 50%	0.447
		*P* = 0.009^c^		*P* = 0.02^c^		*P* = 0.469		*P*<0.0001^c^
	III	0.680	T3	0.232	N2/N3	1.194	>50%	0.364
		*P* = 0.14		*P* <0.0001^c^	–	P=0.42	–	P<0.0001 ^c^
	IV	0.616	T4	0.297	–	–	–	–
		*P* = 0.22		*P* = 0.006^c^	–	–	–	–
Rectum	II	0.228	T2	0.642	N1	1.347	0 to 50%	0.400
		*P*<0.0001^c^		*P* = 0.31		*P* = 0.16		*P*<0.0001^c^
	III	0.470	T3	0.196	N2/N3	1.120	>50%	0.269
		*P* = 0.002^c^		*P*<0.0001^c^		*P* = 0.61		*P*<0.0001^c^
	IV	0.113	T4	0.119	–	–	–	–
		*P*<0.0001^c^		*P*<0.0001^c^	–	–	–	–
Family history								
Sporadic	RC		RC		RC		RC	
Familial	II	1.161	T2	1.462	N1	1.099	N1 ]	1.161
		*P* = 0.54		*P* = 0.36		*P* = 0.65		*P* = 0.44
	III	1.308	T3	1.588	N2/N3	0.963	N2/N3	0.670
		*P* = 0.26		*P* = 0.22		*P* = 0.76		*P* = 0.13
	IV	0.760	T4	1.419	–	–	–	–
		*P* = 0.56		*P* = 0.42	–	–	–	–
Nagelkerke *R*^2^	–	0.121	–	0.132	–	0.058	–	0.088

*AJCC* American Joint Committee on Cancer, *N* node, *RC* reference category, *T* tumor.

^a^AJCC stage I, T1, N0, and 0% mucin are reference groups in analysis of AJCC, T, and N stage, and mucin production, respectively.

^b^Data are presented as odds ratios and *P* values.

^c^Significant.

### Association between gender, age group, tumor location, family history, and nature of surgery

In a univariate analysis of the association between tumor location and the nature of surgery, the only significant result was seen for tumors in the rectum, where there was a much lower risk for having to undergo emergency surgery compared with the cecum (OR = 0.044, *P*<0.0001). The significant result remained when comparing rectal tumors with the right colon (OR = 0.053, *P*<0.0001). In a multivariate analysis, none of the factors gender, age group, or family history were associated with nature of surgery. Location remained a significant factor with OR = 0.054 and *P*<0.0001 for having emergency surgery for a tumor in the rectum compared with the right colon.

### Factor analysis

All the dependent and independent variables could be grouped into seven different factors (components) as shown in Table 4.

**Table 4:** **Factor analysis including both independent and dependent variables studied in relation to colorectal cancer morphology**^**a,b**^

	**Factor (component)**^**c**^						
	**1**	**2**	**3**	**4**	**5**	**6**	**7**
Nature of surgery	0.444	−0.077	−0.135	−0.239	0.418	0.302	−0.348
Gender	0.021	−0.054	0.031	−0.161	0.485	−0.448	0.156
Age group	−0.232	0.006	0.060	0.024	0.098	0.458	0.493
Location	0.014	−0.195	−0.225	−0.119	−0.693^d^	−0.043	−0.028
>1 tumor	−0.017	0.013	−0.049	−0.172	0.225	0.644^d^	0.025
Tumor diameter	0.027	0.216	0.320	0.648^d^	0.150	−0.065	−0.184
AJCC stage	0.758^d^	0.074	−0.030	0.368	0.008	0.145	−0.055
Tumor (T) stage	0.519	0.157	−0.058	0.559	0.208	−0.030	0.008
Node (N) stage	0.754^d^	0.080	0.009	0.173	−0.113	0.101	−0.026
Differentiation	−0.236	−0.159	−0.776^d^	−0.055	−0.025	0.012	0.015
Mucin production	0.001	0.950^d^	0.023	0.025	0.073	0.027	−0.040
Mucin type	0.083	0.926^d^	0.092	0.039	0.103	0.018	0.001
Crohn-like^e^	−0.153	0.048	0.089	0.225	0.657^d^	0.019	−0.021
TILs	−0.207	0.097	0.611^d^	−0.031	0.205	0.017	0.085
Desmoplasia	0.206	−0.023	−0.372	0.471^d^	0.157	−0.044	0.217
Necrosis	0.041	−0.445	−0.008	0.601	−0.021	0.016	0.177
Vascular invasion	0.586^d^	−0.110	0.114	−0.023	0.038	−0.142	−0.002
Perineural invasion	0.609^d^	0.015	−0.041	−0.058	−0.090	−0.134	0.168
Medullary type	−0.027	−0.118	0.837 ^d^	0.049	0.093	0.063	0.046
Budding	0.379	−0.122	0.086	0.041	0.040	0.004	0.683^d^
Tumor margin	0.547^d^	0.101	−0.255	−0.026	−0.119	−0.069	0.356
Family history	0.006	−0.006	−0.084	−0.038	0.128	−0.522^d^	−0.025

*AJCC* American Joint Committee on Cancer, *TILs* tumor-infiltrating lymphocytes.

^a^Variables with loading of >0.40 are usually applied as meaningful loadings on the factor. If a variable has a meaningful loading on more than one component, that variable should be ignored in the interpretation.

^b^A minus (−) before the value indicates a negative correlation.

^c^Factors: 1, Variables related to aggressiveness and extent of tumor spread; 2, factors related to mucin production/mucin type; 3, factors related to microsatellite instability type of colorectal cancer; 4, factors related to tumor size and desmoplastic response to tumor growth; 5, location and peri-tumor lymphocytic infiltration; 6, family history and multiple tumors; 7, budding.

^d^The variable is applied as a meaningful loading on that component.

^e^Crohn-like lymphocytic reaction.

## Discussion

The novel finding of this study is that according to both univariate and multivariate analyses, cases of CRC treated surgically as an emergency are more likely to have multiple tumors. To our knowledge, this has not previously been reported in the literature.

Emergency tumors tended to be of higher AJCC stage (II to IV), T stage (T4), and N stage (N1 to 2/3) which is in line with previous reports [2,7]. This is not unexpected as T stage and AJCC stage reflect the local advancement of the tumor. It seems reasonable that locally advanced tumors, by infiltrating through the bowel wall, could promote perforation. A locally advanced tumor would also be more likely to display vascular and perineural invasion, which, in fact, was seen in our study (OR = 2.086, *P* = 0.001 and OR = 2.500, *P*<0.0001 respectively, in the multivariate comparison). Lymphovascular invasion in turn, would increase the probability of lymph-node metastases, as indicated by the N stage.

Interestingly, there was no difference in mean tumor diameter between the emergency and the elective groups, nor was there any difference in the frequency of mucinous tumors or tumors showing necrosis. Large, mucinous, or necrotic tumors would be expected to be more disposed to causing obstruction or perforation, resulting in emergency surgery. The perforations associated with colon cancer are mainly due to a direct mechanism of local destruction at the site of the tumor, which does not necessarily mean that the tumor itself has to reach a certain size to achieve this destruction. In about one-third of the cases of perforated colon, the perforation is located proximal to the cancer [14]. This is a condition familiar to colorectal surgeons, which is attributed to a diastatic widening of the cecum, eventually leading to perforation. This is often the case in left-sided (sigmoid) tumors. Because of the consistency of the stools in this region, these cancers are prone to cause an obstruction, which in turn leads to dilation of the proximal part of the colon. The law of La Place states that the site of largest diameter requires the least pressure to cause distention. Hence, the cecum is the most vulnerable part of the colon, and will perforate at a certain diameter, usually described as 130 mm in the literature [15] if there is an obstructing distal tumor in the left colon.

**Figure 2:**
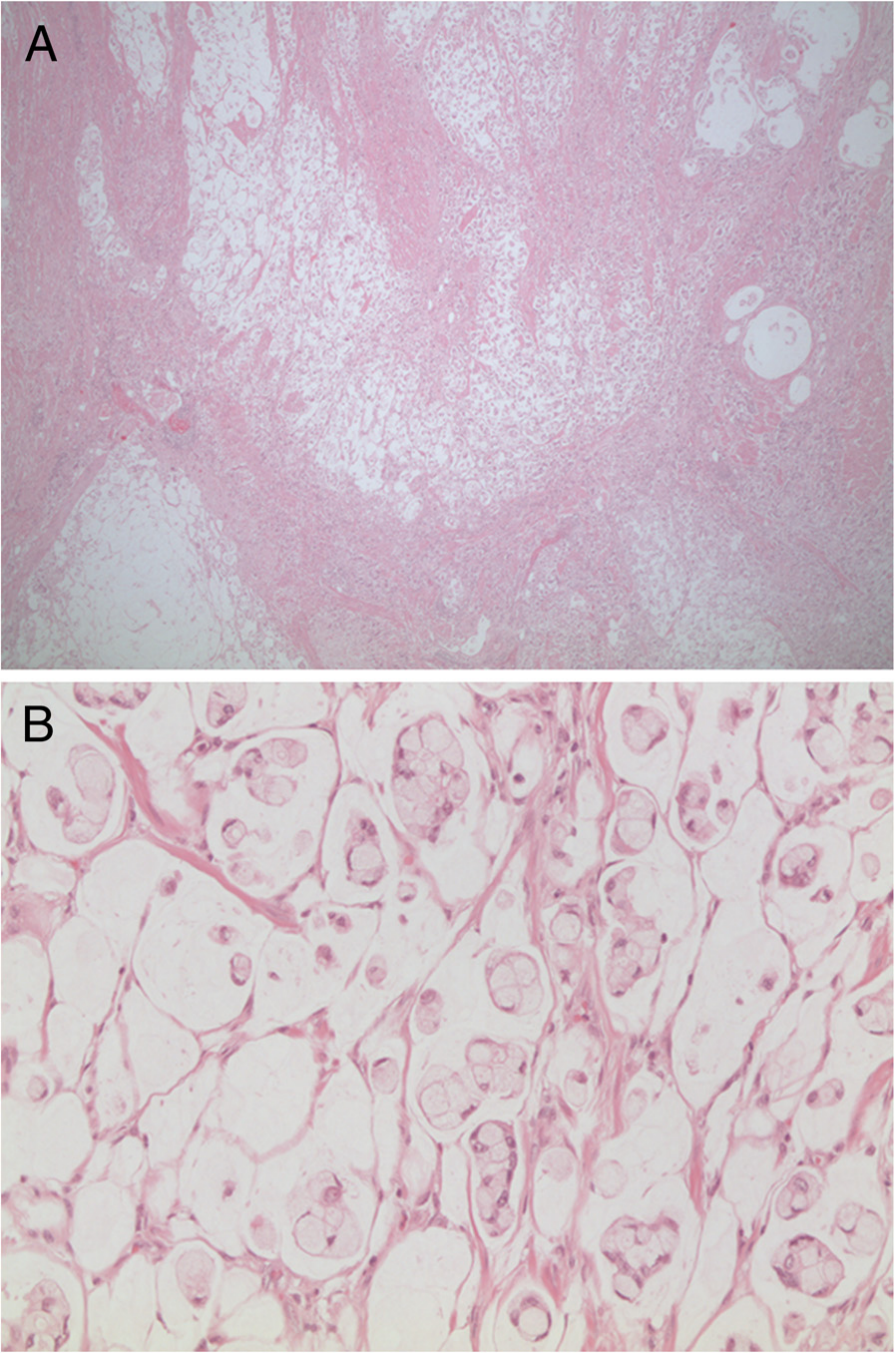
**Poorly differentiated mucinous colorectal cancer (CRC) of the signet-ring cell type.** (**A**) Tumor displaying large dissecting mucus pools filled with tumor cells. (**B**) Same tumor at higher magnification showing signet-ring cells with a large cytoplasmic mucin vacuole and a dislocated nucleus at the periphery. Hematoxylin and eosin, original magnification (**A**) ×25; (**B**) ×200.

Presence of a mucinous tumor with signet-ring cells was more frequent in the emergency group (OR = 3.136, *P* = 0.001 in the multivariate analysis). This type of mucin-producing tumor, with mucin pools filled with cells displaying a large cytoplasmic mucin vacuoles, might make the tumor less cohesive and firm, and thereby more prone to perforation (Figure 2A,B).

Signet-ring cell carcinomas comprise only 0.7 to 2.6% of all CRCs. Compared with other adenocarcinomas, these tumors have a poorer prognosis with higher rates of distant recurrence and lower rates of survival [16].

We found tumors with TILs more than 30/HPF to be less common in the emergency compared with the elective group. A large number of TILs is a distinct feature of the so-called microsatellite instability (MSI)-CRC phenotype, which is seen in most cases of LS and in approximately 12 to 17% of sporadic CRCs. MSI tumors have a unique clinical picture and pathologic phenotype, with a better prognosis and a different response to chemotherapy [16-22]. Approximately 30% of right-sided CRCs are shown to be of the MSI type, and the majority of MSI tumors are located on the right side [23,24]. The most common reported site of obstruction is the sigmoid colon [4], which might explain the under-representation of tumors with a large number of TILs among our emergency cases. Regardless of MSI status, lymphocyte invasion may reflect an anti-tumor immune response [25]. In CRCs treated surgically as an emergency because of perforation, this cellular reaction might not have had time to develop.

Three MSI-associated features, namely multiple tumors, signet-ring cell carcinomas, and a Crohn-like lymphocytic reaction, were more common in the emergency group, whereas a large number of TILs and a circumscribed tumor margin were more common in the elective cases. No difference was seen between the groups for poor differentiation, mucin production, or medullary tumors, which also belong to the MSI spectrum [20]. Thus the ‘MSI-like’ CRC phenotype does not seem to predominate in either emergency or elective cases.

As mentioned above, vascular invasion was more common in the emergency cases in our study. It seems likely that emergency tumors, being more locally advanced, will show a higher frequency of both vascular and perineural invasion. This is reflected in reports showing a worse prognosis for CRCs treated surgically as emergency cases [1,2,5-7]. A higher frequency of vascular invasion should feasibly lead to more distant metastases, but we were unable to assess M stage in our study. A follow-up of our patients over 5 or 10 years could perhaps reveal a correlation between vascular invasion and survival time, as shown in previous studies [26-29].

Finally, the emergency cases also displayed a higher frequency of tumors with an infiltrative margin (OR = 2.452, *P*<0.0001 in the multivariate comparison). This finding is also in accordance with the fact that locally aggressive tumors cause perforation.

We also looked at the effect of gender, age group, tumor location, and family history on the nature of surgical presentation. In a univariate analysis, only tumor location was found to be a significant factor, with a highly significantly lower risk (OR = 0.053, *P*<0.0001) of requirement of emergency surgery for a rectal cancer, compared with a right-sided colon cancer. This significant difference remained in the multivariate comparison (OR = 0.054, *P*<0.0001). This finding is not unexpected and is in line with the clinical picture of rectal cancer and its surgical management.

In the factor analysis, we found that AJCC and N stage were in the same component (factor 1) together with vascular invasion, perineural invasion, and tumor margin. This is not unexpected as these are all features related to the extent of tumor spread and tumor aggressiveness. Mucin production and mucinous type were included in the same component (factor 2). Grade of differentiation (negative correlation with well/moderate differentiation), number of TILs, and medullary type are all features related to the MSI-CRC phenotype (factor 3). Crohn-like peri-tumor lymphocytic infiltrate, which is also an MSI feature, was not included in this factor. Tumor diameter and desmoplasia were grouped together (factor 4). Desmoplastic reaction is generally thought to be a feature favoring the host by encapsulating the tumor, but there are conflicting reports [29]. Factor 5 included tumor location and peri-tumor lymphocytic infiltration. This is in line with our previous observation that the frequency of peri-tumor lymphocytic reaction is higher in right-sided CRCs [13]. Family history and multiple tumors were grouped together (factor 6), and budding separately (factor 7).

One weakness of our study is that it was retrospective for both the enrollment of patients and the histopathologic re-evaluation of tumor tissue. Furthermore, because informed consent was necessary for inclusion, patients who died soon after surgery could not be included. However, this is one of the largest studies on emergency surgery for CRC of its kind. By having a single experienced pathologist re-evaluate all cases, we also avoided the problem of inter-observer variability. Furthermore, we had access to the family history of our patients and thereby were able to relate this information to the nature of surgery.

## Conclusion

Several differences were found between CRCs treated surgically as an emergency and those treated electively. The emergency group had a higher frequency of multiple tumors and a more aggressive histopathologic profile and more advanced stage. Because the distribution of emergency and elective cases was essentially the same between the right and the left colon, the observed differences cannot primarily be attributed to differences in macro-environment or tumor location between the two groups. It is known that emergency colorectal surgery is associated with a poorer outcome and higher recurrence and mortality rates. This has traditionally been considered to be a technical and surgical problem, consequently leading to a more frequent use of adjuvant chemotherapy in such cases. Our study suggests that the complexity of the issue probably involves a more aggressive nature of the tumor itself. If future studies are able to classify the genetic background of these tumors, more precise and adequate colon cancer treatment will become feasible.

## Abbreviations

AJCC: American joint committee on cancer; CRC: Colorectal carcinoma; FAP: Familial adenomatous polyposis; H&E: Hematoxylin and eosin; MSI: Microsatellite instability; N: node; OR: Odds ratio; T: tumor; TILs: Tumor-infiltrating lymphocytes.

## Competing interests

The authors declare that they have no competing interests.

## Authors’ contributions

SG participated in the design of the study, recruitment of the histopathologic tissue, analyses, and interpretation of the data. He performed histopathologic re-evaluations and drafting of the manuscript. EB was mainly responsible for statistical analyses and contributed to reviewing of the draft. AL was the principal investigator in the major ‘Low risk colorectal cancer study’, of which the present study was a spin-off; contributed to the analyses and interpretation of the data, and to the critical review of the draft. UL participated in the conception and design of the study, analysis of the medical records, recruitment of the patients into the elective and emergency groups, interpretation of the results, and critical review of the draft. All authors have approved the final version of the manuscript prior to submission.
